# Oxidative stress and soluble receptor for advanced glycation end-products play a role in the pathophysiology of delirium after cardiac surgery

**DOI:** 10.1038/s41598-021-03007-2

**Published:** 2021-12-08

**Authors:** Jakub Kaźmierski, Piotr Miler, Agnieszka Pawlak, Hanna Jerczyńska, Joanna Woźniak, Emilia Frankowska, Agnieszka Brzezińska, Karina Nowakowska, Katarzyna Woźniak, Michał Krejca, Mirosław Wilczyński

**Affiliations:** 1 Department of Old Age Psychiatry and Psychotic Disorders, Faculty of Gerontology, Medical University of Lodz, Czechoslowacka 8/10, 92-216 Lodz, Poland; 2 Central Clinical Hospital, Medical University of Lodz, Lodz, Poland; 3 CoreLab Central Scientific Laboratory of Medical University of Lodz, Medical University of Lodz, Lodz, Poland; 4 Department of Cardiac Surgery, Central Clinical Hospital, Medical University of Lodz, Lodz, Poland

**Keywords:** Neuroscience, Cognitive neuroscience, Consciousness, Pathogenesis

## Abstract

Coronary-artery bypass graft (CABG) surgery is known to improve cardiac function and decrease mortality, albeit, this method of treatment is also associated with a neuropsychiatric complications including postoperative delirium. The pathophysiology of delirium after cardiac surgery remains poorly understood. Thus, the purpose of this study was to investigate whether oxidative stress reflected by decreased preoperative and postoperative plasma antioxidant activity is independently associated with delirium after cardiac surgery. The second aim was to assess whether decreased antioxidant activity is stress-related or mediated by other pathologies such as major depressive disorder (MDD), anxiety disorders, and cognitive impairment. Furthermore, the putative relationship between pre- and postoperative soluble receptor for advanced glycation end-products (sRAGE) overexpression and plasma antioxidant capacity was evaluated. The patients cognitive status was assessed 1 day preoperatively with the use of the Mini-Mental State Examination Test and the Clock Drawing Test. A diagnosis of MDD and anxiety disorders was established on the basis of DSM-5 criteria. Blood samples for antioxidant capacity and sRAGE levels were collected both preoperatively and postoperatively. The Confusion Assessment Method for the Intensive Care Unit was used within the first 5 days postoperatively to screen for a diagnosis of delirium. Postoperative delirium was diagnosed in 34% (61 of 177) of individuals. Multivariate logistic regression analysis revealed that low baseline antioxidant capacity was independently associated with postoperative delirium development. Moreover, increased risk of delirium was observed among patients with a preoperative diagnosis of MDD associated with antioxidant capacity decreased postoperatively. According to receiver operating characteristic analysis, the most optimal cutoff values of the preoperative and postoperative antioxidant capacity that predict the development of delirium were 1.72 mM and 1.89 mM, respectively. Pre- and postoperative antioxidant capacity levels were negatively correlated with postoperative sRAGE concentration (Spearman's Rank Correlation − 0.198 and − 0.158, p < 0.05, respectively). Patients with decreased preoperative antioxidant activity and those with depressive episodes complicated with lower postoperative antioxidant activity are at significantly higher risk of delirium after cardiac surgery development. sRAGE overexpression may be considered as protective mechanism against increased oxidative stress and subsequent cell damage.

## Introduction

Coronary-artery bypass graft (CABG) surgery is known to improve cardiac function and decrease mortality, however, this method of treatment is also associated with a risk of developing such neuropsychiatric complication as postoperative delirium^1–3^. Depending on cardiac surgery type, postoperative delirium incidence has been reported as high as 72%^4^ with our previous studies revealing the incidence after CABG was 36%^5^. Postoperative delirium syndrome is toxic for neuronal activity metabolism and leads to a long-term cognitive decline, functional deterioration, and poor prognosis^6,7^. Though delirium risk factors are well described, its biological mechanisms are poorly understood preventing from development of adequate and effective delirium prophylaxis and treatment.

It was proved that high amounts of unstable free radicals are formed in the course of cardiac intervention^8^. Such overproduction of reactive oxygen species (ROS) and reactive nitrogen species (RNS) can lead to necrotic cell damage and apoptosis via impaired cellular calcium (Ca(2+)) homeostasis^9,10^. On the other hand, enzymatic and nonenzymatic antioxidants are cellular defense mechanisms that reduce the steady-state concentrations of ROS and RNS and repair oxidative cellular damage^11^.

In cases of increased free radicals production, antioxidant defense measured as antioxidant capacity/activity is crucial for cellular and neuronal injury prevention. In general, antioxidant activity is decreased in conditions associated with oxidative stress, and the administration of chain-breaking antioxidants increases antioxidant capacity^12^. Thus, low total antioxidant activity could be indicative of oxidative stress or increased susceptibility to oxidative damage.

Decreased antioxidative status is characteristic for such pathologies as major depressive disorder (MDD) and cognitive impairment, which are frequent among individuals scheduled for cardiac surgery^13,14^. Furthermore, oxidative stress mediates neuronal injury in the course of cerebral ischemia, Alzheimer’s disease (AD), and Parkinson’s disease^9,15^. A clear inverse association between plasma antioxidant activity and degree of neurological damages induced by ischemic-reperfusion injury is well-established^16^. Moreover, Gariballa et al. study confirmed that ischemic stroke patients had lower levels of blood antioxidant capacity comparing to non-stroke patients^17^. In another study, plasma antioxidant capacity in schizophrenic patients was lower compared to control subjects, and significantly inversely correlated with the severity of symptoms^18^. According to available studies, psychological stress contributes to DNA oxidation and lipid peroxidation, as well as decreases plasma antioxidant capacity^19^. In addition, the association between stress induced by shift work and decreased total antioxidant capacity, and relationship between psychological stress reduction and lower serum lipid peroxide levels were reported^20,21^.

Current studies position soluble receptor for advanced glycation end-products (sRAGE) among cardioprotective agents. Cardioprotective effects of sRAGE may be related to cardiomyocyte apoptosis inhibition via mitochondrial pathways^22^. Moreover, as assessed in experimental studies, RAGE protein levels are significantly higher in the brain tissue of rats which are characterized with increased oxidative stress plasma measures^23^. Recent studies show that sRAGE plays a protective role against the harmful effects of inflammation and free radicals, and raised levels of sRAGE may reflect increased inflammatory response responsible for endothelial lesions and coagulopathy in the course of severe infection^24–26^. As sRAGE is involved in the feedback regulation of the toxic effects of RAGE-mediated signaling, its overexpression can be interpreted as the protective mechanism against cell damage and as a regulator of the receptor synthesis^27^.

The aim of the current study was to investigate whether decreased preoperative and postoperative plasma antioxidant activity and sRAGE levels are independently associated with delirium after cardiac surgery. The second aim was to assess whether decreased antioxidant activity is stress (surgery)-related or mediated by neuropsychiatric pathologies such MDD, anxiety disorders, and cognitive impairment. Furthermore, the putative interaction between pre- and postoperative sRAGE overexpression and plasma antioxidant activity was evaluated.

## Methods

### Overview

The procedures and methodology of the current study regarding anesthesia and surgery, collection of blood samples, statistical analysis, and postoperative delirium assessment were previously used in the study of our design conducted among 123 CABG patients^5^. The present study was approved by the Ethics Committee of the Medical University of Lodz, Poland and was performed in accordance with the ethical standards of the Declaration of Helsinki. The study was conducted in the 14-bed cardiac surgical intensive care unit (ICU) of a university teaching hospital (Central Clinical Hospital, Medical University of Lodz, Poland) between April 2017 and November 2019. The subjects signed an informed consent the day before their operation. The inclusion criteria were: consecutive adult patients scheduled for CABG surgery or CABG surgery with cardiac valve repair or replacement (CVR). Patients who underwent CABG were eligible both in case of on-pump and off-pump surgery, however, the impact of cardiopulmonary bypass (CPB) on the risk of postoperative delirium was controlled in “Statistical analysis”. The exclusion criteria were as follows: concomitant surgery other than CABG or CABG with CVR; preoperative delirium; active alcohol or other substances addiction (abstinence period shorter than 3 months); illiteracy; patients on dietary supplements, and with pronounced hearing and/or visual impairment.

### Preoperative psychiatric and psychological procedures

The study population was examined by a psychiatrist the day prior to the scheduled operation using the Mini-Mental State Examination (MMSE) and Clock Drawing Test (CDT) to assess global cognitive status of participants^28,29^. The MMSE was designed as a rapid screening instrument for cognitive dysfunction. This instrument assesses the following cognitive domains: attention and concentration, memory, language, visuo-spatial skills, calculations, and orientation^28^. The CDT is a nonverbal screening tool in which the patient is asked to draw a clock. The test assesses visual-spatial and planning abilities, long-term attention, memory, auditory processing, motor programming, and frustration tolerance^29^. A diagnosis of MDD and anxiety disorders was established by psychiatrist on the basis of DSM-5 criteria^30^.

### Physical comorbidity

Patients’ medical records and current laboratory tests were screen for diagnoses associated with the physical condition of participants. The presence of hypertension, diabetes, peripheral vascular disease, anemia (haemoglobin concentration < 10 mg/dl), atrial fibrillation, obstructive pulmonary disease, urea and creatinine serum concentration, a history of stroke, ejection fraction measured with echocardiography, the New York Heart Association (NYHA) grade, and Canadian Cardiovascular Society degree were determined and entered into analysis. The EuroScore II and the Syntax Score were calculated with the use of calculators available online (New EuroSCORE II (2011); http://syntaxscore.org/, respectively) and included into analysis.

### Anesthesia and surgery

The procedures and methodology of the current study regarding anesthesia and surgery were previously used in the study of our design conducted among 123 CABG patients^5^. For premedication, midazolam 7.5 mg p.o. a half-hour before the surgery was used. Before induction of anesthesia in all patients, routine monitoring was installed: electrocardiography, invasive arterial blood pressure monitoring, central venous pressure monitoring, cerebral oxygen saturation, peripheral oxygen saturation, and urine output. A standard anesthesia technique was performed in all patients. Induction: fentanyl 5–10 mcg/kg, propofol 1–2.5 mg/kg and rocuronium 0.6–1.0 mg/kg. Medication during maintenance was as follows: fentanyl in continuous intravenous infusion in doses of 2–10 mcg/kg/h, propofol 3–10 mg/kg/h, interrupted doses of rocuronium. Ventilation was provided with a breathing mixture of FiO2 0.5 and air to maintain end-tidal CO_2_ at 35–45 mmHg. From surgical incision to cardiopulmonary bypass connection sevoflurane continuous 0.5–2 vol% was used. In case of hypotension ephedrine in boluses or norepinephrine in infusion was used to counteract profound vasodilation to maintain mean arterial pressure above 55 mmHg. For cardioplegia, DelNido solution was used in all patients. DelNido contains a calcium-free, potassium-rich base solution of Plasmalyte (e.g. Baxter Poland) and an electrolyte composition similar to the extracellular fluid. It also contains lidocaine, mannitol and other additives such a magnesium sulfate. Those constituents serve as a crystalloid component, which is mixed with fully oxygenated whole blood of the patient in a ratio of 4 parts of crystalloid to 1 part of blood. DelNido cardioplegia is administrated as a single dose with an effect that lasts up to 90 min.

After surgery, patients were transferred to the ICU and were placed on mechanical ventilation. Until extubation, they were sedated with morphine in the continuous infusion of 1–2 mg/h and propofol perfusion at a rate 1–2 mg/kg/h. The acceptable levels of arterial blood gases and oxygen saturation > 92% and stabilization of hemodynamic parameters were criteria for extubation which in uncomplicated cases took place 4–8 h after the operation. Patients who underwent CABG or CABG with concomitant valve surgery were operated through a median sternotomy and on cardiopulmonary bypass (CPB) under normothermia. The anterograde DelNido cardioplegia was used for cardiac protection during the operation. In some cases, patients who underwent coronary revascularization were operated without CPB (off-pump CABG-OPCAB), on a beating heart, either through the median sternotomy or through left-sided mini-thoracotomy. In CABG patients both internal thoracic artery (ITA) and saphenous vein grafting (SVG) were performed (between 1 and 2 ITA grafts, and 1 and 3 SVG grafts).

In the group of CABG patients concomitant with valve surgery, 14 of them had aortic valve replacement (AVR) and only three mitral valve replacement (MVR).

### Measurement of serum antioxidant capacity and sRAGE levels

The procedures and methodology of the current study regarding collection of blood samples were previously used in the study of our design conducted among 123 CABG patients^5^. The venous blood samples were taken twice during the study period: the day prior to the surgery (baseline measurement) and on the first postoperative day, between the hours 07:00 and 09:00 a.m. The blood samples were centrifuged at 7000 rpm for 10 min and were frozen at − 80 °C until biochemical parameters were determined. The serum total antioxidant activity and sRAGE levels were measured with ELISA Kit and an Antioxidant Assay Kit (Cayman Chemical, USA for antioxidant activity; BioVendor, Czech Republic for sRAGE).

The antioxidant assay relies on the ability of antioxidants in the sample to inhibit the oxidation of ABTS (2,2′-Azino-di-(3-ethylbenzothiazoline sulphonate)) to ABTS·+ by metmyoglobin. The produced amount of ABTS·+ can be monitored by reading the absorbance at 405 nm. Under the reaction conditions used, the antioxidants in the sample cause suppression of the absorbance at 405 nm to a degree that is proportional to their concentration. The capacity of the antioxidants in the sample to prevent ABTS oxidation is compared with that of Trolox, a water-soluble tocopherol analogue, and is quantified as millimolar Trolox equivalents.

Protocols were performed according to the manufacturer’s instructions. The concentration of the protein in patient sample was determined by interpolation from the standard curve. For washing steps the Stat-Matic Plate Washer II (Sigma-Aldrich) was used. The absorbance was read by Multifunctional Microplate Reader VICTORTM X4 (Perkin Elmer, USA). All ELISA results were analyzed with WorkOut 2.5 Software and the mean concentration of protein per ml was determined by referring to the four parameter logistic (4-PL) curve.

The tests were conducted by investigators that were blinded to clinical data.

### Delirium diagnosis

The procedures and methodology of the current study regarding postoperative delirium assessment were previously used in the study of our design conducted among 123 CABG patients^5^. Following surgical interventions, the Confusion Assessment Method for the Intensive Care Unit (CAM for ICU) and the Memorial Delirium Assessment Scale (MDAS) with the cut-off score 10 were used to diagnose delirium^31,32^. Each individual was assessed by the study psychiatrist once a day within the first 5 days after surgery. Before each administration of the CAM for ICU Unit, the level of sedation/arousal was assessed using the Richmond Agitation Sedation Scale (RASS)^33^. If the patient was deeply sedated or was unarousable which corresponds to − 4 or − 5 on the RASS, evaluation was stopped and repeated later. If the RASS was above − 4 (− 3 through + 4), assessment with the CAM for ICU was administered. If there was an inconsistency between the diagnostic tools regarding the delirium diagnosis, the final consensus was established within the study team physicians collecting information from all available sources. Nursing and medical staff were interviewed and/or clinical notes were interrogated for mention of delirium or delirium symptoms.

### Statistical analysis

The methodology of the current study regarding statistical analysis was previously used in the study of our design conducted among 123 CABG patients^5^. Quantitative variables are expressed as medians and interquartile ranges (IQRs). For categorical variables, the number of observations (n) and fraction (%) were calculated.

Normality was tested using the Shapiro–Wilk’s test for normality. Differences between two independent samples for continuous data were analyzed using the Mann–Whitney U test (since the distributions of variables were different from normal). The effect size for continuous variables was measured with the rank-biserial correlation coefficient.

For categorical variables, statistical analysis was based on the chi-squared test or Fisher’s exact test. Cramer’s V coefficient was calculated to assess the effect size for categorical variables. Spearman’s rank correlation coefficients were calculated to assess the correlation between two quantitative variables. Nonparametric analysis of variance of aligned rank transformed data (ART) was used to compare the levels of markers in different groups of patients (taking into account two qualitative factors). Partial eta-squared was calculated as the effect size measure. The minimum study sample size was calculated using the power analysis, estimating the expected effects from our previous studies and assuming an alpha level of 0.10 and a power of 80% (minimum sample size for each group is 37 patients). Initially, baseline and perioperative variables were evaluated for univariate association with postoperative delirium. For quantitative variables (preoperative and postoperative antioxidant activity), significantly associated with the occurrence of delirium, receiver operating characteristic (ROC) curves were drawn (Area Under Curve with Standard Error was given) and optimal decision thresholds (based on the Youden’s index value) were found. The sensitivity, specificity, positive predictive value, and negative predictive value were calculated. Odds ratios with 95% confidence intervals were also presented. Factors significant in univariate comparisons (p < 0.10) were included in a forward stepwise logistic regression model to identify the set of the independent risk factors for delirium. The results were considered significant for p < 0.05. All of the calculations were performed using STATISTICA (version 13.3, 2017; StatSoft, Inc., Tulsa, OK, USA) and the R-project (the rcompanion package).

### Ethics approval and consent to participate

The study was approved by the Ethics Committee of the Medical University of Lodz, Poland. The subjects signed an informed written consent for the participation in the study.

## Results

### Basic findings

**Figure 1:**
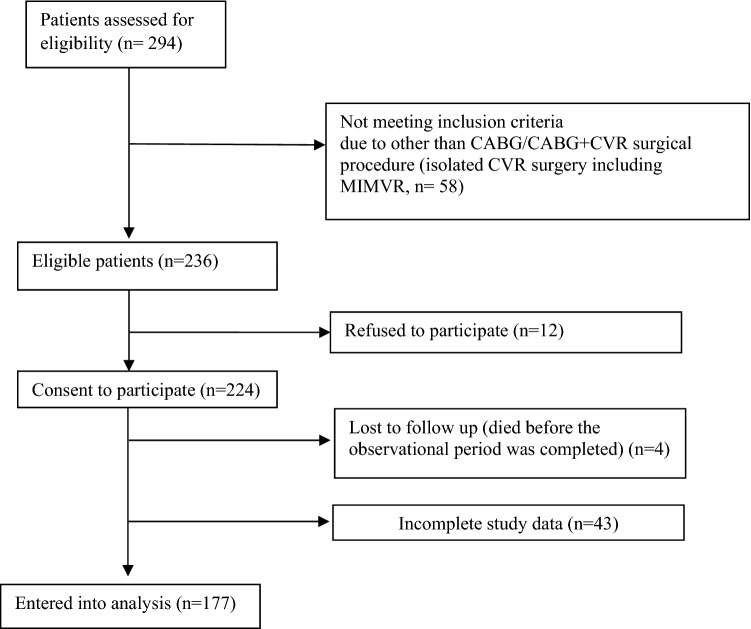
Number of patients excluded and included in the study analysis. *CABG* Coronary-artery bypass graft, *CVR* Cardiac valve replacement, *MIMVR* Minimally Invasive Mitral Valve Repair.

Elective cardiac surgery was performed in two hundred ninety four patients during the study period; of these, 58 subjects did not meet the inclusion since they underwent different than CABG/CABG plus CVR surgery (isolated CVR surgery without CABG including minimally invasive mitral valve repair [n = 58]), and 12 individuals did not sign an informed consent (Fig. 1).

Of the 224 patients who signed their informed consent and were enrolled, four patients were lost to follow-up since they died before the observational period was completed, and 43 individuals had incomplete study data (these patients were not included in the analysis due to failure during samples collection or inappropriate samples collection (coagulation), n = 28; and due to incomplete postoperative delirium evaluation, n = 15). The remaining 177 patients were entered into the analysis. All the patients had chronic coronary syndromes. Baseline demographic characteristics and patients’ comorbidities are presented in Table 1. The majority of patients (n = 145, 82%) had preoperative EuroScore II between 0 and 2.99, the score between 3 and 5.99 was calculated in 21 (12%) individuals, and the score of 6 and more in 11 (6%) patients. Postoperative delirium developed in 34% (61 of 177) of patients. Patients with postoperative delirium had a significantly longer stay in the ICU (4 (IQR: 1–18) vs. 3 (IQR: 2–7) days; p < 0.001) and a longer total duration of hospitalization (14 (IQR: 8–60) vs. 10 (IQR: 6–50) days; p < 0.001) compared with patients who did not develop delirium.

**Table 1:** Demography characteristics and co-morbidities of the study participants.

	N	%
**Demography**		
Age (years)	67	(63–71)
Gender male	138	77.97%
**Years of education**		
Between 1 and 7 years	39	22%
Between 8 and 11 years	109	62%
12 or more years	29	16%
Living area		
City > 100.000 people	74	42%
City < 100.000 people	60	34%
Country	30	24%
Social status		
Living with family	157	88.7%
Living alone	19	10.7%
Other	1	0.6%
**Psychiatric comorbidities and characteristics**		
Depression	33	18.6%
Anxiety disorders	14	8.0%
MMSE score	28	(25–29)
CDT score	7	(5–9)
**Cardiac surgery type, physical comorbidities and characteristics**		
CABG + valve replacement	17	9.6%
OPCAB	44	25%
Anaemia (haemoglobin concentration < 10 mg/dl)	27	15%
Urea concentration (mmol/l)	6.7	(5.5–7.8)
Creatinine concentration (µmol/l)	84.8	(74–99)
Peripheral vascular disease	30	17%
Atrial fibrillation	22	12%
Arterial hypertension	145	81%
Diabetes	61	34%
History of cerebral infarct	21	11.9%
Ejection fraction (%)	52	(44.5–58)
hs Troponin T the day before surgery (ng/l)	16.5	(9.5–32.5)
hs Troponin T day 0 (after surgery) (ng/l)	279.74	(132–332.5)
hs Troponin T the day after surgery (ng/l)	274.6	(164.5–469.5)
Median EuroScore II	1.29	(0.85–1.80)
Syntax score ≤ 22^a^	27	17%
Syntax score 23–32^a^	61	38%
Syntax score ≥ 33^a^	72	45%
NYHA		
0	5	2.8%
I	15	8.5%
II	108	61%
III	48	27.1%
IV	1	0.6%
CCS		
I	21	11.9%
II	78	44.1%
III	69	39%
IV	9	5.0%

For continuous variables, the medians and interquartile ranges (IQRs) are given; for categorical variables, the number of observations (n) and fraction (%) were calculated.

*CABG* Coronary-artery bypass graft, *CCS* Canadian Cardiovascular Society degree, *CDT* Clock Drawing Test, *hs* high sensitive, *MMSE* Mini-Mental State Examination, *NYHA* New York Heart Association grade, *OPCAB* Off-pump coronary artery bypass.

^a^The Syntax score was calculated for 160 CABG patients excluding subjects with concomitant valve surgery.

### Univariate and multivariate comparisons

The findings of the univariate analysis of factors related to the comorbidities, biomarkers, anesthesia, and surgical procedures are shown in Tables 2, 3, and 4.

**Table 2:** Antioxidant activity levels and variables related to demography and mental condition of patients analyzed in univariate analysis.

Variable	Non-delirious^a^ (n = 116)	Delirious^a^ (n = 61)	Effect size^b^	p
Age (years)	66 (61–69)	70 (66–72)	− 0.340	**< 0.001**
Gender female	15 (13.0%)	24 (39.0%)	0.303	**< 0.001**
Depression	9 (7.8%)	24 (39.0%)	0.385	**< 0.001**
Anxiety disorders	5 (4.3%)	9 (14.7%)	0.184	**0.02**
Alcohol addiction^c^	8 (6.9%)	5 (8.2%)	0.024	0.768
Preoperative AA (µmol/l)	2.38 (1.85–3.08)	1.32 (1.04–2.31)	0.443	**< 0.001**
Postoperative AA (µmol/l)	2.11 (1.42–2.94)	1.37 (0.90–1.89)	0.409	**< 0.001**
Preoperative sRAGE	0.81 (0.61–1.01	1.06 (0.79–1.43)	− 0.382	**< 0.001**
Postoperative sRAGE	0.75 (0.58–1.03)	1.09 (0.71–1.44	− 0.365	**< 0.001**

Significant values are in [bold].

*AA* antioxidant activity, *sRAGE* soluble receptor for advanced glycation end-products.

^a^For continuous variables, the medians and interquartile ranges (IQRs) are given; for categorical variables, the number of observations (n) and fraction (%) were calculated.

^b^For continuous variables rank-biserial correlation coefficient was calculated; for categorical variables Cramer’s V coefficient was presented.

^c^Only patients with at least 3 months abstinence were included.

**Table 3:** Variables related to physical condition of patients analysed in univariate analysis.

Variable	Non-delirious^a^ (n = 116)	Delirious^a^ (n = 61)	Effect size^b^	p
Peripheral vascular disease	13 (11.2%)	17 (27.9%)	0.211	**0.005**
Arterial hypertension	89 (76.7%)	56 (91.8%)	0.186	**0.013**
NYHA	2 (2–2)	2 (2–3)	− 0.175	**0.029**
AF^c^	10 (8.6%)	12 (19.7%)	0.159	**0.034**
Ejection fraction (%)	58 (46–68)	46 (40–58)	− 0.233	**0.096**
Diabetes	35 (30.0%)	26 (42.6%)	0.125	**0.098**
Urea concentration (mmol/l)^c^	6.8 (5.5–7.6)	6.7 (5.4–8.0)	0.018	0.849
Creatinine concentration (mcmol/l)^c^	83.7 (75.4–98.3)	88 (68.1–104.8)	− 0,028	0.758
Anaemia^c,d^	16 (13.8%)	11 (18.0%)	0.056	0.456
Cerebrovascular disease^c^	12 (10.3%)	9 (14.7%)	0.065	0.464
COPD	6 (5%)	5 (8.25)	0.060	0.516
CCS	2 (2–3)	2 (2–3)	0.115	0.503
Syntax score ≤ 22^e^	20 (18.5%)	9 (17.3%)	0.076	0.500
Syntax score 23–32^e^	39 (36%)	18 (34.6%)	0.070	0.543
Syntax score ≥ 33^e^	49 (45.4%)	25 (48%)	0.085	0.489
Median EuroScore II	1.11 (0.83–1.92)	1.47 (0.94–2.01)	0.114	0.523
hs Troponin T the day before surgery (ng/l)	15.0 (8.5–30.5)	18.0 (10–34)	0.023	0.342
hs Troponin T day 0 (after surgery) (ng/l)	259.74 (121–312)	300 (221–423)	0.034	0.123
hs Troponin T the day after surgery (ng/l)	254.2 (164.5–469.5)	294.8 ((211–387.2)	0.36	0.243

Significant values are in [bold].

*AF* atrial fibrillation, *CCS* Canadian Cardiovascular Society Degree, *COPD* Chronic Obstructive Pulmonary Disease, *NYHA* New York Heart Association grade.

^a^For continuous variables, the medians and interquartile ranges (IQRs) are given.

^b^For continuous variables rank-biserial correlation coefficient was calculated; for categorical variables Cramer’s V coefficient was given.

^c^Preoperative variables.

^d^Haemoglobin concentration < 10 mg/dl.

^e^The Syntax score was calculated for 160 CABG patients excluding subjects with concomitant valve surgery. Among these patients, 52 (32.5%) were delirious.

**Table 4:** Variables related to anaesthesia, surgery and postoperative condition of patients analysed in univariate analysis.

Variable	Non-delirious^a^ (n = 116)	Delirious^a^ (n = 61)	Effect size^b^	p
CABG with cardiac valve replacement	8 (6.9%)	9 (14.75%)	0.127	**0.092**
ECC	81 (69.8%)	52 (85%)	0.170	**0.024**
Hyperthermia^d^	9 (7.8%)	10 (16.4%)	0.133	**0.078**
Na < 130 mEq	5 (4.3%)	6 (9.8%)	0.145	**0.021**
Aortic cross-clamping^c^ (min.)	40 (30–55)	43 (30–70)	− 0.114	0.270
Duration of surgery (h)	4.0 (3–4.5)	4.0 (4–4.5)	− 0.085	0.350
Circulatory support^c^	2 (1.7%)	1 (1.6%)	0.003	0.97
Corticosteroids use^c^	0 (0%)	1 (1.6%)	0.104	0.345
pCO2 ≥ 45^d^ (mmHg)	24 (20.7%)	18 (29.5%)	0.099	0.19
pO2 ≤ 60^d^ (mmHg)	18 (15.5%)	13 (21.3%)	0.072	0.33

Significant values are in [bold].

*CABG* Coronary-artery bypass graft, *ECC* extracorporeal circulation.

^a^For continuous variables, the medians and interquartile ranges (IQRs) are given.

^b^For continuous variables rank-biserial correlation coefficient was calculated; for categorical variables Cramer’s V coefficient was given.

^c^Intraoperative variables.

^d^Postoperative variables.

The unadjusted risk of postoperative delirium was higher both for patients with decreased preoperative and postoperative antioxidant activity (rank-biserial correlation = 0.443; p < 0.001, rank-biserial correlation = 0.409; p < 0.001; respectively). Furthermore, pre- and postoperative sRAGE levels were significantly higher in delirium group comparing to non-delirium subjects (rank-biserial correlation = − 0.382; p < 0.001, rank-biserial correlation = − 0.365; p < 0.001; respectively).

Patients who had decreased baseline preoperative antioxidant activity remained at increased risk of developing delirium after controlling for the following variables significant in univariable analysis: age, female gender, weight, depression and anxiety disorders, NYHA grade, hypertension, diabetes, peripheral vascular disease, atrial fibrillation (AF), and the presence of the extracorporeal circulation (ECC) (Table 5). Raised sRAGE concentrations did not enter the final multivariate logistic regression model.

**Table 5:** Factors independently associated with delirium after cardiac surgery revealed in multivariate stepwise logistic regression analysis.

Variables	Coefficient	Standard error	OR (95% CI)	p
Depression^a^	2.641	0.570	14.027 (4.592–42.852)	0.000
Antioxidant capacity^a^	− 0.821	0.225	0.440 (0.283–0.684)	0.000
Gender female	1.798	0.482	6.039 (2.348–15.530)	0.000
Age	0.098	0.033	1.103 (1.035–1.177)	0.003
Atrial fibrillation^a^	1.304	0.568	3.684 (1.211–11.208)	0.022
ECC	1.234	0.551	3.435 (1.167–10.112)	0.025
Constant	− 7.708	2.361	–	0.001

The regression model is statistically significant: χ^2^ = 41,285, df = 6, p < 0.001; Hosmer–Lemeshow test: χ^2^ = 12.185, p = 0.143; Nagelkerke R^2^ = 0.503.

*ECC* extracorporeal circulation.

^a^Preoperative variables.

### Optimal biomarkers thresholds and correlations with other analyzed variables

According to the ROC analysis, the most optimal cutoff value of preoperative antioxidant capacity that predicts the development of delirium was 1.72 mM, with sensitivity of 65.6% and specificity of 78.4%, positive predictive value of 0.61 and negative predictive value of 0.81 (OR = 6.93; 95% CI 3.48–13.81) (area under the curve = 0.722; standard error = 0.043; 95% CI 0.64–0.80; p < 0.001). The median preoperative and postoperative antioxidant capacity levels in the whole population were 2.13 mM (IQR: 1.29–2.91) and 1.84 mM (IQR: 1.22–2.65), respectively. Nonparametric analysis of variance of aligned rank transformed data (ART) did not reveal significant differences in pre- and post-operative antioxidant activity between men and women, more advanced age, subjects undergoing CABG vs. CABG plus CVR surgery, on-pump vs. off-pump surgery, individuals with and without anxiety disorders. There were also no significant interactions between AA and comorbidities (hypertension, peripheral vascular disease, and diabetes). However, ART analysis showed a significant interaction between depression and delirium with regard to the postoperative antioxidant activity (partial eta-squared = 0.025). According to the post hoc pairwise comparisons, median postoperative antioxidant capacity was decreased among patients with preoperative MDD and postoperative delirium when compared to patients without depression and delirium (1.63 mM; IQR: 1.13–2.54 vs. 2.19 mM; IQR: 1.49–2.97; p = 0.05). The above observation seems to be associated with a diagnosis of MDD rather than delirium since pairwise comparisons did not reveal differences in postoperative antioxidant activity between individuals without depression and without delirium, and those without depression who developed delirium (p = 0.17). ART analysis showed that women and patients with depression had significantly higher preoperative sRAGE levels (p < 0.001 for females and MDD) and postoperative sRAGE levels (p = 0.004 for females and p = 0.005 for MDD) comparing to men and non-depression subjects, despite delirium occurrence. Preoperative sRAGE levels were also significantly increased in patients with anxiety disorders (p = 0.047) and a diagnosis of peripheral vascular disease (p = 0.04). In addition, ART analysis showed a significant interaction between depression and delirium with regard to the preoperative sRAGE concentration (partial eta-squared = 0.028). There was no significant correlation between sRAGE levels and other comorbidities (diabetes, hypertension). According to the post hoc pairwise comparisons, median preoperative sRAGE levels were increased among patients with preoperative MDD and postoperative delirium when compared to patients without depression and delirium (1.16 ng/ml; IQR: 0.92–2.07 vs. 0.8 ng/ml; IQR: 0.60–1.02; p = 0.009). However, in that case, the above observation seems to be delirium-related, since pairwise comparisons revealed significant differences regarding preoperative sRAGE concentration between individuals without depression and without delirium, and those without depression who developed delirium (p = 0.03). Interestingly, significant negative correlations between pre- and postoperative antioxidant activity and postoperative sRAGE levels were observed (Spearman’s rank correlation coefficients − 0.198 and − 0.158, respectively; p < 0.05).

There were no significant relationships between pre- and post-operative antioxidant activity and age (Spearman’s rank correlation coefficients: − 0.099, p = 0.19; − 0.147, p = 0.07, respectively), MMSE score (0.037, p = 0.62; 0.057, p = 0.45, respectively), CDT score (0.004, p = 0.96; 0.067, p = 0.37, respectively), duration of surgery (− 0.021, p = 0.78; 0.023, p = 0.76, respectively), duration of aortic cross-clamping (− 0.041, p = 0.64; 0.025, p = 0.77, respectively) and intubation time (− 0.13, p = 0.07; − 0.087, p = 0.25, respectively). There was significant correlation between pre- and post-operative sRAGE levels and age (Spearman’s rank correlation coefficients: 0.148, p = 0.049; 0.169, p = 0.024, respectively) and postoperative sRAGE concentration and intubation time (Spearman’s rank correlation coefficient: 0.215, p = 0.004).

There were no significant correlations between pre- and post-operative sRAGE and MMSE score (− 0.026, p = 0.70; − 0.015, p = 0.83, respectively), CDT score (− 0.077, p = 0.30; 0.020, p = 0.78, respectively), duration of surgery (0.043, p = 0.57; 0.069, p = 0.36, respectively), and duration of aortic cross-clamping (0.076, p = 0,381; 0.047, p = 0.59, respectively), and preoperative sRAGE level and intubation time (0.120, p = 0.11).

## Discussion

In this prospective study, we investigated the association between pre- and postoperative total plasma antioxidant capacity, and the risk of postoperative delirium. Moreover, the putative interaction between pre- and postoperative sRAGE overexpression and plasma antioxidant activity was evaluated.

The incidence of post-surgery delirium in the current study was 34% compared with the incidence rate of 11–50% reported in the available literature and our previous study of a similar design (36%)^3,5,34–37^. In the current study, the prevalence of psychiatric comorbidities was 18.6% for MDD and 8% for anxiety disorders. These numbers are consistent with the prevalence of psychiatric disorders in CABG patients reported in other studies (15–20% for MDD and 7.6–10% for anxiety disorders)^38,39^. According to the multivariate regression model, patients with lower preoperative antioxidant capacity were more likely to develop delirium, and this association was independent of age, gender, MDD diagnosis, physical comorbidity, and the presence ECC.

Studies investigating risk factors of postoperative delirium have heterogenous findings. This inconsistency of the results may be, in part, related to the multifactorial etiology of delirium. It may be hypothesized that different mechanisms which contribute to delirium act through the same final pathways. This might explain the heterogeneity of research findings.

Our previous study devoted to delirium pathogenesis revealed that patients with MDD and increased levels of cortisol prior to surgery are more likely to develop postoperative delirium^5^. Secondly, patients with increased hypothalamic–pituitary–adrenal (HPA) axis reactivity secondary to pathologies such as MDD were characterized with higher postoperative cortisol levels compared with patients without MDD and, possibly as a consequence, were more likely to develop postoperative delirium.

Current biomedical knowledge indicates also that MDD and other neuropsychiatric pathologies may be associated with oxidative stress and its biochemical consequences^40^.

Patients with depression are at higher risk of cardiovascular disease (CVD), obesity, diabetes, cancer and cognitive impairment, they have also a higher all-cause mortality rate^41^.

It is possible that metabolic dysfunctions and excessive cellular ageing may be underlying mechanisms that contribute to this poorer physical health in patients with depression^42^.

In recent meta-analysis, the association between depression and two oxidative stress markers (8-hydroxy-2-deoxyguanosine (8-OHdG) and F2-isoprostanes (IsoPs)) was investigated^40^. The analysis revealed that both 8-OHdG (10 studies, 1308 subjects) and F2-isoprostanes (8 studies, 2471 subjects) are increased in depression. In another meta-analysis of 115 articles, lower total antioxidant activity was noted in patients with severe depressive episodes^43^. Studies have also confirmed an increase of other oxidative stress biomarkers—malondialdehyde (MDA) and 4-hydroxy-2-nonenal (4-HNE) levels in the brains of patients with AD and mild cognitive impairment (MCI)^44^. Moreover, increased levels of IsoPs have been detected in the cerebrospinal fluid of AD patients^44^, and the level of F2-IsoPs in the ventricular fluid was negatively correlated with the brain weight^45^.

According to available studies, the 8-OHdG marker is significantly increased in AD individuals as compared to control subjects^46^ Interestingly, 8-OHdG appears to precede all the typical hallmarks of AD, such as neurofibrillary tangles and Aβ plaques, and specifically occurs decades before Aβ aggregation in AD patients^44^.

It should be stressed that patients with CVD are characterized with increased oxidative DNA damage and decreased antioxidative status comparing to healthy subjects, which in turn may contribute to oxidative stress-related neuropsychiatric pathologies^47^. Current results indicate that MDD prior to surgery is strongly and independently associated with postoperative delirium. Interestingly, ART analysis showed significant interaction between depression and delirium with regard to the postoperative antioxidant activity (partial eta-squared = 0.025). According to the post hoc pairwise comparisons, median postoperative antioxidant capacity was decreased among patients with preoperative MDD and postoperative delirium when compared to patients without depression and delirium. The above observation seems to be associated with a diagnosis of MDD rather than delirium, since pairwise comparisons did not reveal differences in postoperative antioxidant activity between individuals without depression and without delirium, and those without depression who developed delirium. Since as indicated in regression analysis, MDD prior to surgery increases the risk of postoperative delirium, lower postoperative antioxidant activity may be a factor that mediates the impact of depression on postoperative cognitive status.

On the contrary, our analysis revealed that patients with decreased antioxidant activity measured the day prior to surgery are at increased risk of delirium, even after controlling for depression, age, gender, and variables related to the physical condition of participants.

The putative mechanism responsible for oxidative stress-related neuronal damage was described in previous studies^48^. Unstable free radical species attack cells causing damage to lipids, proteins, and DNA which can initiate a chain of events leading to disease onset.

Living organisms developed an antioxidant system to counteract free radicals and prevent tissue damage. This system includes such enzymes as superoxide dismutase and catalase, and macromolecules such as albumin and ceruloplasmin. Both endogenous and food-derived antioxidants represent the total antioxidant capacity which is reduced in conditions characterized with increased oxidative stress. Low antioxidant capacity is characteristic for depression. Genetic polymorphisms, poor appetite, psychological stressors, metabolic syndrome, sleep disorders, substance abuse which are frequently present in MDD may also contribute to it^44^. In the current study, we also investigated the association between the expression of the receptor of advanced glycation end products (RAGE) and postoperative delirium as RAGE is found to play an important role in the development of CVD, cognitive impairment and AD, and is known to be involved in microvascular and macrovascular complications in diabetes^49,50^. RAGE is characterized as a multiligand receptor and, apart from AGEs, RAGE interacts with other ligands, such as the S100 proteins, high mobility group box 1, and amyloids. Ligand binding increases RAGE activity which mediates proinflammatory responses and generates oxidative stress that may contribute to the pathogenesis of CVD, neuronal degeneration, and Aβ accumulation, which in turn induce cognitive impairment^51^. There are also recently described truncated forms of RAGE, including C-terminally truncated soluble form of RAGE (sRAGE) which consists of spliced and shredded variants^52^. These heterogeneous forms of sRAGE bind ligands including AGEs and can antagonize RAGE signaling in vitro and in vivo. It is possible that the attenuated plasma sRAGE seen in individuals with metabolic dysfunction may contribute to AD due to reduced capacity to scavenge RAGE ligands and attenuate RAGE signaling.

In the current study, sRAGE expression was higher after surgery when compared to baseline values, and both pre- and post-operative sRAGE levels were increased in patients who developed delirium comparing to non-delirium subjects. Moreover, there were significant negative correlations between pre- and post-operative antioxidant activity and postoperative sRAGE levels. These may reflect the protective mechanisms of sRAGE according to which, overexpression of sRAGE regulates inflammation and reduces cell damage related to oxidative stress. In this model, patients with lower antioxidant status were more likely to develop delirium and had a higher peak of protective plasma sRAGE levels after surgical intervention. Cardioplegic arrest and the ischemic period during cardiac surgery constitute the serious load for the heart and brain cells. In this context, the type of cardioplegic solution used plays an important role in cardiac and brain protection. Crystalloid cardioplegia provides myocardial protection and a bloodless and motionless operative field after single-dose application; whereas intermittent administration of blood-based cardioplegic (BBC) solution may give better myocardial protection especially for its high oxygen and metabolites-carrying capacity^53^. In the current study, all patients received DelNido cardioplegia which combines the features of crystalloid and BBC solutions (see the Method section).

## Strengths and limitations of the study

The advantages of the present study include its prospective design, a considerable number of recruited participants, multifactorial analysis of variables related to both mental and physical condition of participants, as well as intraoperative and postoperative delirium risk factors. The study includes in-depth statistical analysis to enable proper investigation of the mutual associations between biomarkers, and postoperative delirium.

The limitations of the current report should be also noted. Unfortunately, our analysis did not include such potential risk factors of delirium as malnutrition, the use of multiple medications or medications with a high cholinergic index. Furthermore, plasma concentrations of antioxidant activity measured in blood samples may not directly reflect the central nervous system condition and activity. Moreover, oxidative DNA damage biomarkers were not assessed, which together with antioxidant capacity measure could investigate oxidative state in more detailed manner. On the other hand, antioxidant capacity is a marker which gives very relevant biological information compared to that obtained by the measurement of individual components, as it considers the cumulative effect of all antioxidants present in plasma and body fluid (superoxide dismutase, catalase, and glutathione peroxidase; macromolecules such as albumin, ceruloplasmin).

## Conclusions

The current study revealed that decreased preoperative antioxidant activity and major depressive disorder independently predispose to postoperative delirium development. Lower postoperative antioxidant activity was not significant in the final multivariate regression model, however, was significantly decreased among patients with preoperative MDD and postoperative delirium comparing to patients without MDD and delirium. Therefore, it can be hypothesized that lower postoperative antioxidant activity may be a factor that mediates the impact of depression on postoperative delirium. In addition, both pre- and postoperative sRAGE levels were increased in patients who developed delirium comparing to non-delirium subjects, and significant negative correlations between pre- and postoperative antioxidant activity and postoperative sRAGE levels were observed. These may reflect the protective mechanisms of sRAGE according to which, overexpression of sRAGE regulates inflammation and reduces cell damage related to oxidative stress.

## Data Availability

The datasets used and/or analysed during the current study are available from the corresponding author on reasonable request.
